# Properties of the PVA-VAVTD KOH Blend as a Gel Polymer Electrolyte for Zinc Batteries

**DOI:** 10.3390/gels7040256

**Published:** 2021-12-10

**Authors:** Alisson A. Iles Velez, Edwin Reyes, Antonio Diaz-Barrios, Florencio Santos, Antonio J. Fernández Romero, Juan P. Tafur

**Affiliations:** 1 School of Chemical Science and Engineering, Yachay Tech University, Yachay City of Knowledge, Urcuqui 100650, Ecuador; alisson.iles@yachaytech.edu.ec (A.A.I.V.); edwin.reyes@yachaytech.edu.ec (E.R.); adiaz@yachaytech.edu.ec (A.D.-B.); 2 Grupo de Materiales Avanzados para la Producción y Almacenamiento de Energía, Universidad Politécnica de Cartagena, Aulario II, Campus de Alfonso XIII, 30203 Cartagena, Spain; florencio.santos@upct.es

**Keywords:** gel polymer electrolyte, PVA blend, potassium hydroxide, XRD and ATR-FTIR, TGA-DTG, ionic conductivity, cyclic voltammetry study, Zn–air battery

## Abstract

Rechargeable zinc-air batteries are promising for energy storage and portable electronic applications because of their good safety, high energy density, material abundance, low cost, and environmental friendliness. A series of alkaline gel polymer electrolytes formed from polyvinyl alcohol (PVA) and different amounts of terpolymer composed of butyl acrylate, vinyl acetate, and vinyl neodecanoate (VAVTD) was synthesized applying a solution casting technique. The thin films were doped with KOH 12 M, providing a higher amount of water and free ions inside the electrolyte matrix. The inclusion of VAVTD together with the PVA polymer improved several of the electrical properties of the PVA-based gel polymer electrolytes (GPEs). X-ray diffraction (XRD), thermogravimetric analysis (TGA), and attenuated total reflectance- Fourier-transform infrared spectroscopy (ATR-FTIR) tests, confirming that PVA chains rearrange depending on the VAVTD content and improving the amorphous region. The most conducting electrolyte film was the test specimen 1:4 (PVA-VAVTD) soaked in KOH solution, reaching a conductivity of 0.019 S/cm at room temperature. The temperature dependence of the conductivity agrees with the Arrhenius equation and activation energy of ~0.077 eV resulted, depending on the electrolyte composition. In addition, the cyclic voltammetry study showed a current intensity increase at higher VAVTD content, reaching values of 310 mA. Finally, these gel polymer electrolytes were tested in Zn–air batteries, obtaining capacities of 165 mAh and 195 mAh for PVA-T4 and PVA-T5 sunk in KOH, respectively, at a discharge current of −5 mA.

## 1. Introduction

Polymer electrolytes have been considered as a possible ionically active material since Fenton and coworkers discovered the poly(ethylene oxide) (PEO) complexes with alkali metal ions [1] and their potential application in batteries was discovered by Armand [2]. Polymer electrolytes (PEs) have been drawing attention because they are a safer choice than liquid electrolytes [3]. In recent decades, numerous electrolytes containing different cations such as Zn(II) [4,5], Cd(II) [6], Cu(II) [7], or Co(II) [8] have been investigated as samples of thin-film polymeric electrolytes. Nevertheless, the main drawback of PE materials is their relatively low ionic conductivity at room temperature [9,10]. Ionic conductivity increases in inverse proportion to the degree of crystallinity and the viscosity of the polymeric matrix [11]. Therefore, many efforts have been implemented to improve the ionic motion of PEs.

Thus, gel polymer electrolytes (GPEs) were proposed to improve the limited ionic conductivity of common solid PEs. GPEs are also known as plasticized PEs, and they are neither liquid nor solid. Therefore, they have properties of both, conserving the cohesive characteristics of solids together with the ion diffusive properties of liquids [12]. Usually, a polymer containing heteroatoms such as oxygen, nitrogen, or sulfur [2,12,13] acts as a hosting matrix of inorganic salts of Li^+^ or Zn^2+^ to obtain the gel polymer electrolyte.

On the other hand, the GPEs applied in energy storage devices need to have high ionic conductivities, good mechanical properties, and excellent electrochemical stability at room temperature. Metal-air batteries consist of a metal as a negative electrode coupled to an air-breathing positive one, and are considered as alternatives to the conventional Li-ion batteries used nowadays [14,15] because metal-air batteries provide higher energy density thanks to the oxygen involved in the reaction being directly drawn from the surrounding air—it is not stored in the battery in advance [15,16]. Notably, alkaline zinc-air batteries, among metal-air batteries, hold enough merit to be highlighted. These devices use relatively inexpensive and environmentally friendly raw materials and provide high specific energy values (1084 Wh/kg) [17]. In addition, their low-cost, high natural abundance, low toxicity, nonflammability, and large stable potential window make them very attractive as energy storage applications [17,18,19].

According to the literature, polyvinyl alcohol (PVA) has been widely used due to its good film-forming ability, good mechanical strength, optical properties, biocompatibility, biodegradability, and non-toxicity [20,21]. PVA is a semicrystalline, hydrophilic, synthetic polymer material at room temperature, which has been extensively used as a host material in various PEs systems due to its polar nature, easy processability, non-corrosive nature, and low production cost [22]. Some researchers have highlighted the electrochemical and mechanical behavior of PVA upon the addition of differently concentrated salt solutions and blending with other materials to prepare GPEs. Saeed et al. [23] reported the effect of a high ammonium salt concentration on electrolytes-based PVA, and they reached an ionic conductivity of 5.17 × 10^−5^ S/cm at room temperature. Tiong et al. [24] reported that the highest ionic conductivity reached by the PEO/PVA blend-based gel polymer electrolytes was 5.5 × 10^−3^ S/cm. In addition, Merle et al. [25] improved the ionic conductivity values until 2.2 × 10^−1^ S/cm by crosslinking the PVA-KOH polymer with poly(ethylene glycol) diglycidyl ether.

Moreover, vinyl acrylic emulsions are important classes of polymer dispersions usually applied in architectural coating [26]. One of the most important industrial latexes is the emulsion copolymerization of vinyl acetate (VA)/butyl acrylate (BA). According to their properties, VA and BA present glass transition temperatures at T_g(VA)_ = 32 °C and T_g(BA)_ = −54 °C, which give a stable thermal range to provide a suitable temperature for electrochemical applications [24,25].

In addition, the KOH incorporation inside GPE membranes provides free ions, which interact with the polar groups of PVA, modifying their structural arrangement and improving the motions of the ions inside the electrolyte matrix. Lewandowski et al. [27] and Santos et al. [4] reported ionic conductivity in the order of 10^−3^–10^−1^ S/cm for PVA-KOH-H_2_O alkaline polymer electrolytes, respectively. In this work, different GPEs are prepared from PVA blended with VAVTD as the host matrix and doped with a concentrated KOH solution used as a source of ions to improve the system’s ionic conductivity. The structural, electrical, and transport properties of the synthesized membranes have been analyzed as a function of polymer blend proportions and the results point to a good material to be applied as GPE in Zn batteries.

## 2. Results and Discussion

### 2.1. Structural Characterization

#### 2.1.1. Swelling Ratio

**Figure 1:**
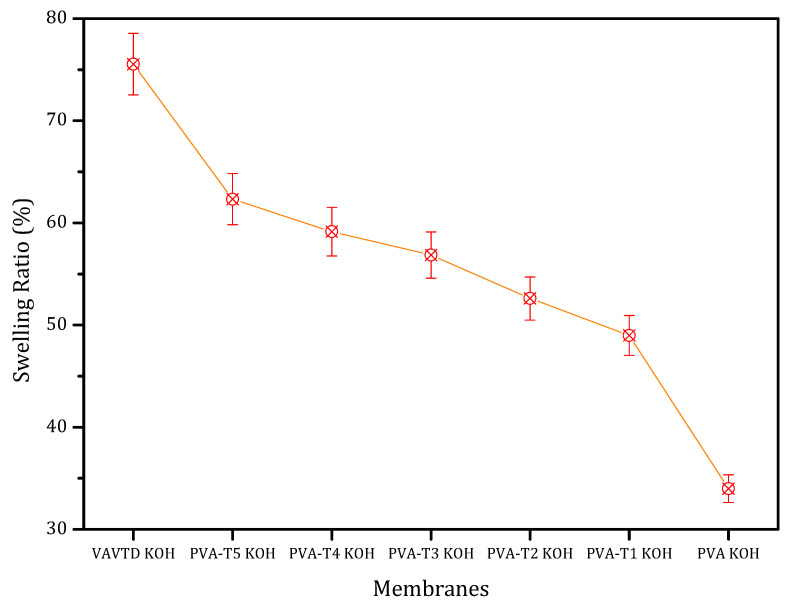
Swelling ratio of the PVA-VAVTD KOH matrix as a function of the VAVTD increase in membrane.

PVA-VAVTD membranes synthesized in this article were soaked in a 12 M KOH solution to provoke the entrance of KOH and water inside the polymeric matrix and thus improve their electrical properties. The swelling ratio (SR) was determined for all GPEs following Equation (2). It was observed that the progressive increase in VAVTD in the PVA matrix improved the swelling ratio of the resulting matrix as shown in Figure 1.

Compared with the KOH-doped PVA matrix, it was possible to observe that a minimum swelling ratio was established for the PVA KOH system. In addition, pure VAVTD reached the highest SR value—75%—which means that it retained three times its electrolyte weight. The progressive incorporation of VAVTD elevated the overall swelling ratio of the matrix due to the growth of internal sites. Although VAVTD absorbed a large quantity of KOH, its mechanical resistance was significantly affected and the resulting membrane did not keep good properties for being used as a GPE. It was thus necessary to add another polymer, namely PVA, to improve the mechanical resistance. However, VAVTD was used to increase the KOH absorption resulting in an excellent blend.

#### 2.1.2. XRD Analysis

**Figure 2:**
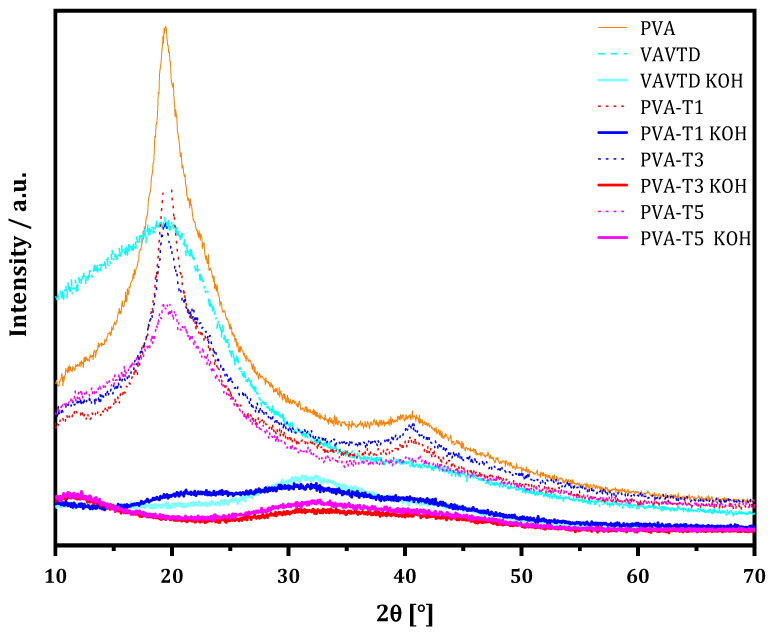
XRD patterns of PVA-VAVTD and PVA-VAVTD KOH systems at different VAVTD contents.

The XRD spectra were analyzed in order to determine the VAVTD influence on the crystalline structure of the PVA matrix. The XRD profile of pure PVA, VAVTD, PVA-VAVTD, and PVA-VAVTD KOH samples are depicted in Figure 2, where VAVTD was introduced to decrease the PVA crystallinity due to the disruption of internal hydrogen bonds generated by (OH)^–^ arrangement. The XRD pattern of the pure PVA membranes revealed a crystalline peak at 2θ = 19.8° and a shoulder at 23.01°, representing reflections from (101) and (200) from a monoclinic unit cell [28]. Similar studies have considered polymer blending as an effective technique to reduce PVA crystallinity [27,28,29].

**Figure 3:**
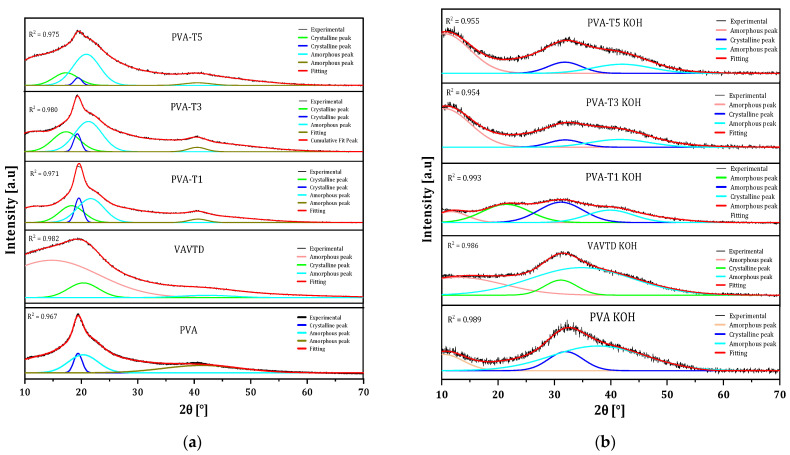
Comparison of the deconvoluted XRD patterns of (**a**) PVA-VAVTD and (**b**) PVA-VAVTD KOH systems at different VAVTD contents.

On the other hand, the XRD profile of VAVTD was presented with the deconvolution method in Figure 3a. This showed the peak at 2θ = 20°, but with a high level of amorphousness compared with pure PVA. This peak was related to the presence of VA segments [30] long enough to form nanocrystals due to the highest participation of VA in the VAVTD synthesis.

Moreover, the deconvoluted XRD spectra of PVA KOH, VAVTD KOH, and PVA-blend KOH systems are shown in Figure 3b. It is notable the modification of the PVA peaks, such as the reduction of the principal crystalline peak at 2θ = 20° and the wideness of peak at 2θ = 40° [31], when the VAVTD content increases. This crystalline reduction was caused by the breaking of hydrogen bonds formed between –OH groups, provoking the intensity reduction seen in the comparison spectra of Figure 2. Thus, these wider and lower intensity peaks confirm the amorphousness of the PVA blends’ structure [32].

As can be seen, pure PVA deconvolution presents a crystalline peak at 20°, which is consistent with previously reported results [31,32]. This peak was observed again when VAVTD was included in the membrane, but in this case, another crystalline peak was observed due to the presence of VAVTD (Figure 3a). Similar behavior has previously been observed for PVA-based composites [31,32]. The inclusion of KOH and water inside the polymeric matrix provokes important structural changes providing very different XRD patterns and, thus, new deconvolution peaks (Figure 3b). However, the intensity of these peaks is very low, as can be seen in Figure 2.

From the deconvolution method, the *Xc* of each PVA blend system was obtained using Equation (3), and all data are summarized in Table 1, where it can be seen that *Xc* decreases with the amount of VAVTD and the inclusion of KOH and water. Thus, the electrolyte with the most amorphous region corresponds to PVA-T5 when KOH is inserted, with *Xc =* 5.06%. These results confirm the favored amorphous nature of the polymer electrolytes. In addition, it is notable that, in PVA-VAVTD GPEs, the area of the crystalline peak (1 0 1) decreases as the VAVTD content increases, and this peak entirely disappears when the membranes are immersed in KOH (Figure 3b). Moreover, the degree of crystallinity of PVA and PVA KOH matches with the values reported in the literature [31,33].

#### 2.1.3. ATR-FTIR Analysis

**Figure 4:**
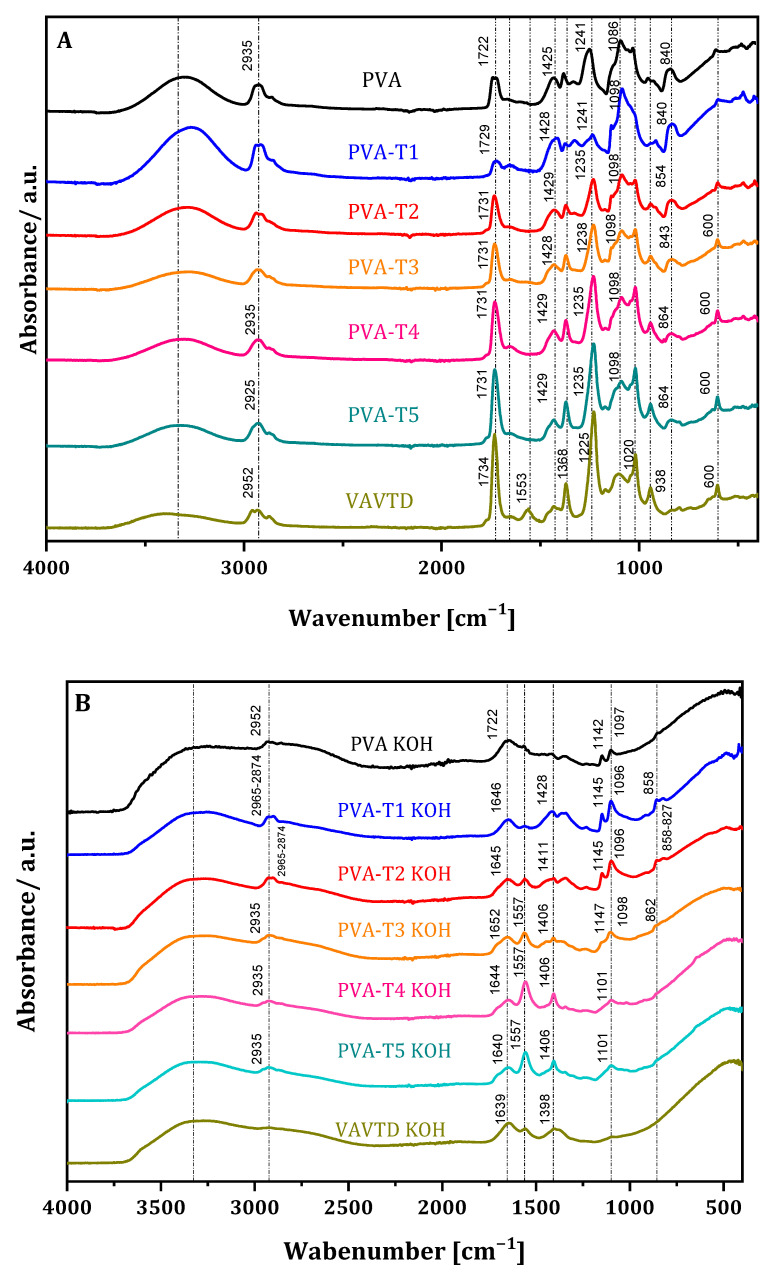
Comparison of the ATR-FTIR spectra of (**A**) PVA, VAVTD, PVA-VAVTD; and (**B**) PVA-VAVTD KOH membranes at different VAVTD proportions.

The ATR-FTIR spectra (Figure 4A) show the characteristic bands of PVA and VAVTD polymers, as well as of PVA-VAVTD blends. Regarding the PVA structure, bands associated with the C–O, C–O–C and C=O vibrational motions of acetate groups were identified at 945–1086, 1241 and 1722 cm^−1^, respectively. The VAVTD spectrum shows the same characteristic bands, but they are shifted to 1020, 1225 and 1734 cm^−1^, respectively. In addition, there is no C=C band at 1600–1680 cm^−1^, which corroborates both polymers’ complete polymerization [29]. All of the spectra show an intense band between 3100 and 3500 cm^−1^, which corresponds to the (OH)^–^ stretching vibration. The bands at 2925 and 2884 cm^−1^ arise from the stretching of CH_3_–and–CH_2_–groups [34].

With regard to PVA-VAVTD systems (PVA-T), the spectra display similar vibrational frequencies for all of them, although differences in their intensities were found. Furthermore, the combination of the PVA and VAVTD terpolymer can be appreciated because the characteristic absorption bands of both polymers are preserved. In addition, the interaction between the two polymeric chains is confirmed by the shift in the main bands. PVA-T1 presents a similar ATR-FTIR spectrum to that of PVA, but the increase in the VAVTD amount inside the polymer provides spectra close to that of VAVTD terpolymer.

On the other hand, the incorporation of KOH and H_2_O molecules inside the PVA-VAVTD polymeric matrix broke its semicrystalline structure and induced several changes in the ATR-FTIR spectra (Figure 4B). The band at υ 1645 cm^−1^ confirms the bending mode frequency of water [35]. In addition, υ 1733, 945 and 1241–1225 cm^−1^ related to carboxylate, C–O and C–O–C vibrational motions, disappeared at all spectra, which may confirm the hydrolysis of acetyl groups by (OH)^–^ groups of the KOH [4]. On the other hand, a new band is observed at 1569 cm^−1^, which may be assigned to the asymmetrical stretching vibration absorption of –C=O–(–O–K), as it has already been reported [4,31]. In fact, the oxygen atoms of –OH and the carbonyl groups have lone pairs available to coordinate with K^+^ ions and form C=O–K^+^/C–O–K^+^ complexes [4].

Furthermore, the intensity of this band increased with the amount of VAVTD in the blend due to the higher number of carboxylate groups included in the polymeric matrix. Finally, the peak at 1143 cm^−1^ associated with the C–O stretching mode is mostly attributed to the remaining crystallinity of the PVA because it is able to form some domains [32]. This band depends on the new intermolecular hydrogen bondings that the samples can build, which were lost for PVA-T3 KOH, PVA-T4 KOH and PVA-T5 KOH membranes showing the dominance of the amorphous region as the VAVTD content increases.

The fundamental –OH absorption region presents a broader peak between 3100 and 3500 cm^−1^ associated with hydrogen interactions. Additionally, the PVA-VAVTD KOH blend has wider and more intense bands than PVA-VAVTD, indicating a higher amount and stronger hydrogen bonds inside the polymeric matrix. Furthermore, the introduction of KOH and water molecules produces changes in the chain network, breaking some H-bonds and forming new H-interactions between the hydroxyl anions, water, and acetate groups of the polymer chains.

In this section, it is worth noting that the synthesis of the polymer blend, casting, and its immersion in KOH solution was performed under ambient conditions without any atmosphere control. Thus, CO_2_ from the air could always be in contact with the blend. However, it is known that the KOH solution is used to capture CO_2_ from the air, generating K_2_CO_3_ [36]. Furthermore, PVA-KOH GEPS previously synthesized by our group had to be prepared in a controlled atmosphere to prevent carbon dioxide forming during the casting and when the membrane was immersed in KOH 12 M, preventing the formation of carbonates inside the polymer [4]. The presence of carbonate in the electrolyte is a concern for Zn/air batteries because it can form K_2_CO_3_, which may be deposited on the air electrode, blocking the oxygen transfer and resulting in the earlier performance decline of the Zn/air battery [37]. Additionally, the Zn electrode may also be passivated by precipitating insoluble ZnCO_3_ on the electrode surface [38].

**Figure 5:**
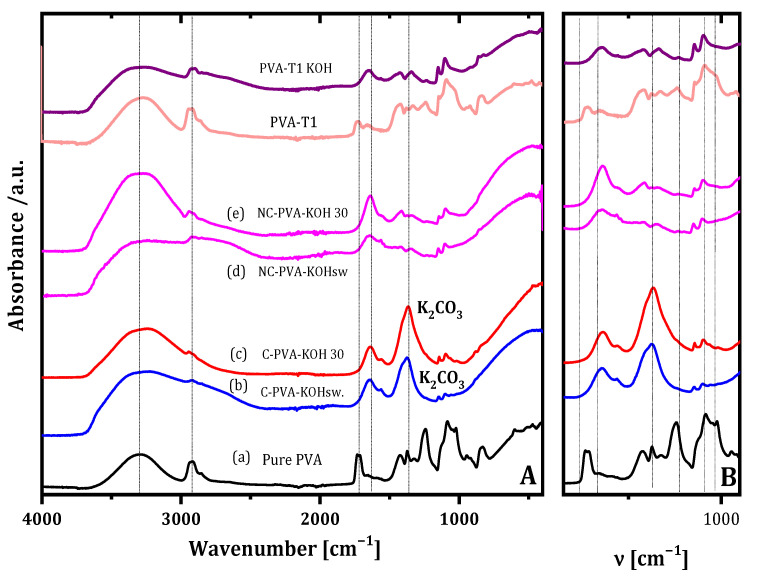
(**A**) ATR-FTIR spectra of pure PVA (**a**) PVA-KOH (**b**) and PVA-KOH immersed in 12 M KOH solution (**c**) synthesized in the presence of carbon dioxide; PVA-KOH (**d**) and PVA-KOH immersed in 12 M KOH solution (**e**) synthesized in the absence of carbon dioxide; and PVA-T1 and PVA-T1 KOH membranes synthesized in the presence of carbon dioxide; (**B**) depicts the K_2_CO_3_ vibration region. Sw indicates that PVA-KOH was swelled in KOH 12 M solution.

Figure 5 shows the ATR-FTIR spectra of PVA-KOH and PVA-KOH immersed in 12 M KOH solution, synthesized in the presence and in the absence of carbon dioxide. As can be seen, when CO_2_ is present, an intense peak assigned to K_2_CO_3_ was observed at υ 1370 cm^−1^ [39]. This band appeared in the spectra of both PVA-KOH and that soaked in KOH 12 M membranes. However, this band disappeared for membranes synthesized when the atmosphere was controlled, avoiding the absorption of CO_2_.

Contrarily, PVA-VAVTD and PVA-VAVTD soaked in KOH 12 M solution were synthesized in the presence of atmospheric CO_2_, but the ATR-FTIR spectra do not show the 1370 cm^−1^ peak. Furthermore, this is a clear advantage of the PVA-VAVTD-KOH blend with respect to the PVA-KOH ones.

#### 2.1.4. Thermal Analysis

**Figure 6:**
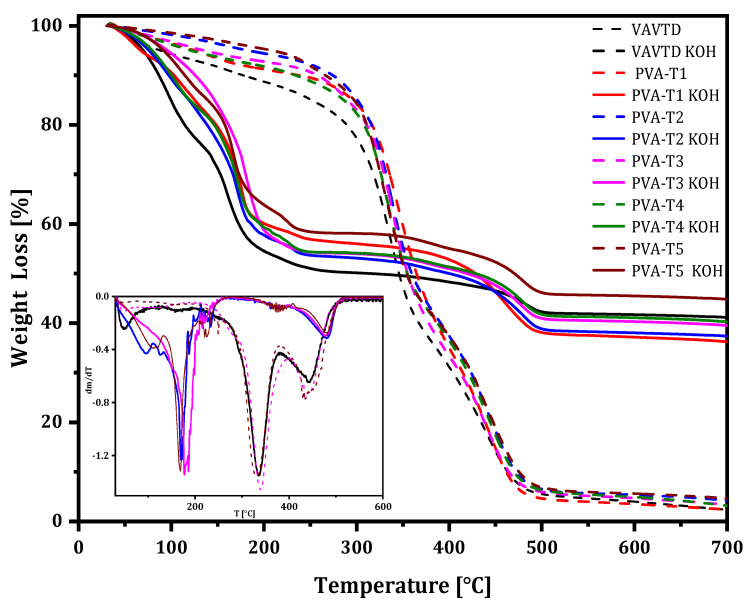
TG curves of PVA-VAVTD and PVA-VAVTD KOH membranes. Ins: DTG curves of electrolytes.

Figure 6 compares the TGA analysis obtained from pure VAVTD, PVA-VAVTD, and PVA-VAVTD soaked in the KOH solution [4,40,41]. The VAVTD terpolymer (black line) presents three degradation steps. The first one occurs in the water region due to the slow loss of internal water, which suppose a 10% of weight loss. The second degradation step starts at ~300 °C until ~380 °C, where 60% of the mass is lost. The last degradation step occurs at ~420 °C and is associated with the breaking backbone. Finally, at ~480 °C, a stable behavior is observed, with a remaining residue of ~5% wt. Similar behavior has been reported for PVA [4,36] and PVA with BA/VAc [42] or PMMA blends [43].

PVA-VAVTD blends show TGA curves very close to those of VAVTD and PVA polymers, and they do not show changes with the VAVTD amount included in the polymeric matrix. However, PVA-VAVTD KOH displays very different TGA curves indicating strong structural changes. This behavior is very similar to the results obtained for PVA-KOH soaked in 12 M KOH solution [4]. The first mass loss occurs between 30 °C and 150 °C, which is associated with the elimination of water. Then, it reaches a loss of 20 wt%, 10% higher than those of VAVTD and VAVTD-KOH films, indicating the presence of more water inside the polymer due to the swelling of the membrane which occurred during its immersion in KOH solution.

A second weight loss occurs with an onset at 150 °C. It is worth noting that this temperature onset is much lower than that observed for VAVTD-PVA membranes, at ~300 °C, confirming the lower thermal stability of the membranes due to KOH solution uptake. This fact is clearly shown in the dm/dT vs. T graph included in the inset of Figure 6, where the peaks corresponding to the second step are found at 342 °C for PVA-VAVTD and at 170 °C for PVA-VAVTD KOH membranes. It should be noted that the TGA curves of PVA-VAVTD KOH are independent of the VAVTD quantity included in the GPEs. This fact points to the fact that the shift to lower temperatures may be associated with the high entrance of KOH and water [44], which interacts with (OH)^–^ and C=O groups which are mainly present in PVA chains. Furthermore, these interactions can explain the reduction in thermal stability observed for membranes soaked in KOH solution, agreeing with the higher amorphous behavior observed in XRD.

The entrance of water molecules during the swelling process was confirmed by the increasing weight loss at a temperature lower than 150 °C. Additionally, the entrance of KOH inside the polymer was demonstrated with the residue obtained at 700 °C. As can be seen, the GPEs preserve 40% of their original mass, which can be associated with the formation of K_2_O by the decomposition of KOH [45] according to Equation (1):

(1) $$2{\mathrm{KOH}}_{\left(s\right)}\to {\;K}_{2}O_{\left(s\right)}+{\;H}_{2}O_{\left(g\right)}$$

Once again, it has to be noted that the percentage of weight remaining at 700 °C is the same for all PVA-VAVTD KOH GPEs, independently of the amount of VAVTD inside the polymer. Thus, KOH should mainly be included in the PVA portion of the polymer.

As a result, the TGA analysis confirms how incorporating KOH and H_2_O molecules inside the GPEs contributes to the weakening of the thermal resistance due to their interactions with the pendant groups of the membranes. This result supports the ATR-FTIR and XRD analyses. When the KOH-H_2_O penetrates the hydrophilic GPEs matrix due to PVA segments, it increases the amorphous behavior and the distance between the polymer chains, providing more free volume for molecular movement [44], decreasing its melting temperature, and allowing a more accessible ionic motion and transport.

### 2.2. Electrochemical Characterization

#### 2.2.1. The Influence of VAVTD Content on Ionic Conductivity

Ionic conductivity is an important factor in determining the applicability of a membrane as a polymer electrolyte in batteries. The gel polymer electrolytes used in this study are composed of a host polymer, VAVTD or PVA-VAVTD, soaked in 12 KOH solution to incorporate (OH)^–^, K^+^, and water molecules inside the polymeric matrix. Furthermore, the amount of KOH and water inside the polymer host will determine the number of ionic carriers, and thus, the ionic conductivity values.

**Figure 7:**
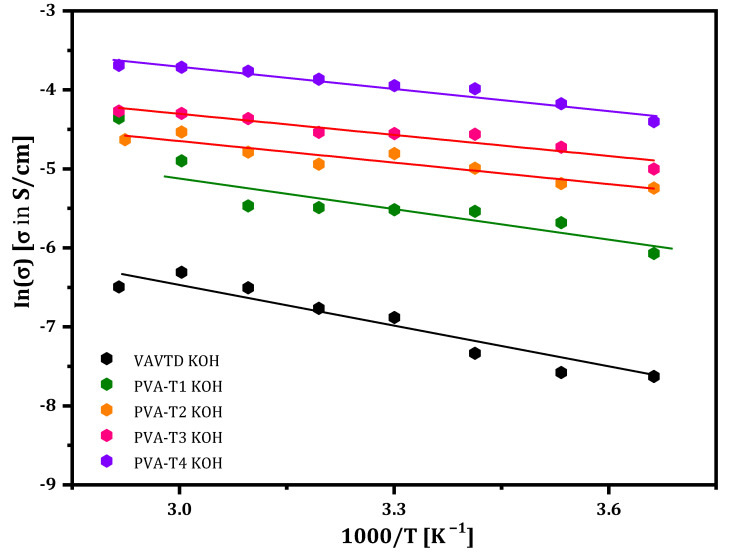
Ionic conductivity of PVA-based electrolytes at different temperatures.

Figure 7 shows the dependence of ionic conductivity with the temperature for GPEs based on PVA at different concentrations of VAVTD and soaked in KOH solution. The ionic conductivity values were obtained from Equation (4). The plots follow a linear fitting, confirming the Arrhenius behavior, as can be deduced from Equation (5), proving that the conductivity is thermally assisted [46].

**Figure 8:**
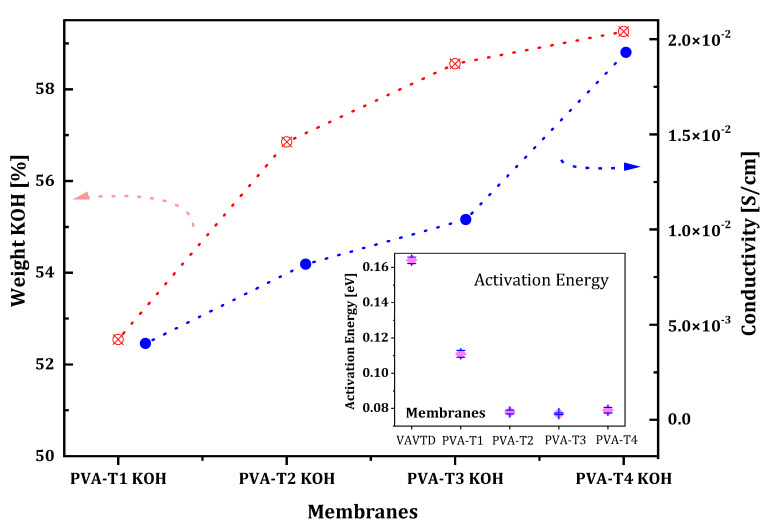
Comparison between the ionic conductive values and KOH absorption percentage (SR) of PVA-VAVTD-based electrolytes.

From the slope of the linear fitting, the Activation Energy (Ea) values for the GPEs studied can be calculated, which are shown in the inset of Figure 8. As can be seen, Ea values are higher for GPEs with a low amount of VAVTD, but this reaches a constant value of ~0.033 eV for the membrane with a higher VAVTD quantity.

Table 2 and Figure 1 demonstrate that PVA-VAVTD polymers have a higher swelling ratio, i.e., they absorb a larger amount of KOH and water, with the increase in VAVTD quantity added to the PVA homopolymer. In addition, conductivity values follow the same tendency of increasing their value with the amount of VAVTD. This fact indicates that more KOH and water molecules are stabilized inside the polymer matrix due to the interaction with a higher number of acetate groups of the VAVTD, as was deduced from ATR-FTIR results. Thus, the ionic conductivity values increase as a consequence of the higher ionic concentration inside the polymer as well as the greater amorphousness and the higher chains separation, as previously deduced. Figure 8 shows the increase in the swelling ratio and conductivity values with the amount of VAVTD inside the blend.

As confirmed by XRD analysis, the crystallization of the PVA-VAVTD GPE membrane was broken by doping with the KOH solution. In addition, this effect improves when the temperature increases, generating a more structural relaxation of the polymer chains (amorphous phase) and expanding the free volume. As a result, this decrease the energy barrier to ionic transport and promotes the fast ion migration [47]. The maximum ionic conductivity, 1.93 × 10^−2^ S/cm, was found when the VAVTD content was 80 wt%, and it reduced to 4.0 × 10^−3^ S/cm when the VAVTD content was 50 wt%. Table 2 summarizes the ionic conductivity and Ea values for VAVTD and the PVA-VAVTD KOH analyzed.

The conductivity of PVA-VAVTD systems could be explained by the mechanism of ions transferring through polymer molecular chains, implying an association–dissociation process between the ions and polar groups of the PVA [47,48] and acrylic groups of the VAVTD matrix, presumably following a Grotthuss mechanism, as has been described before for PVA-KOH GPEs [4]. This phenomenon can be attributed to the increase in the swelling ratio when the VAVTD content increases, as shown in Table 2. This means that a higher amount of KOH and H_2_O molecules penetrates the polymer, favoring the ionic conductivity. In addition, the increase in ion mobility inside the electrolyte is related to the segmental motion of the chains that creates a large free volume and improves the pathway of ionic species. In addition, all blends of PVA-VAVTD KOH have lower Ea values than the VAVTD KOH membrane, which decrease with the amount of VAVTD included in the GPE, until reach a minimum value of ~0.077 eV. This fact indicates that membranes with a lower amount of VAVTD restrict ion mobility, whereas in GPEs with higher VAVTD contents, the energy necessary for providing the ionic movement decreases. The spatial arrangement changes of the electrolytes were demonstrated by the XRD and ATR-FTIR techniques, removing the crystalline domains and favoring the amorphous region.

It was also observed that the membranes of VAVTD pure have lower ionic conductivity and the highest activation energy. However, they present a higher KOH absorption than the other membranes shown in Table 2. This apparent contradictory result is explained by the poor mechanical resistance of pure VAVTD film. This result proves that the VAVTD terpolymer is a poor electrolyte, and it cannot work alone, making necessary the inclusion of PVA to form a sufficiently mechanically resistant blend.

#### 2.2.2. Cyclic Voltammetry

**Figure 9:**
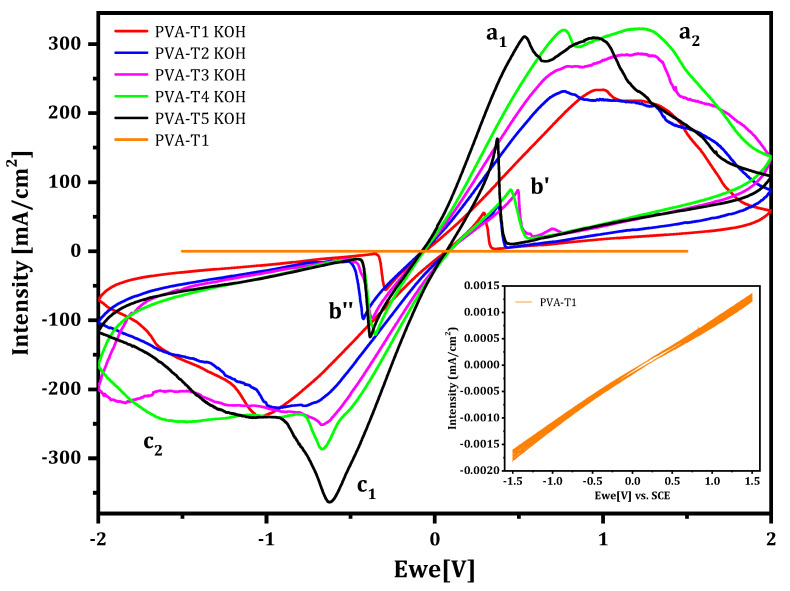
Comparison of the cyclic voltammetry of PVA-VAVTD and GPEs immersed in KOH 12 M at different VAVTD contents. Inset: cyclic voltammetry of PVA-T1 membrane. Peak labels are explained in the text.

Cyclic voltammetry analysis was performed using a Zn/GPE/Zn cell to confirm the ionic transport in the GPEs. Voltammograms of PVA-VAVTD (inset) and PVA-VAVTD-KOH GPEs with different contents of VAVTD are shown in Figure 9. The cyclic voltammograms of PVA-VAVTD membranes without immersion in KOH salt show capacitive curves which are due to the absence of mobile ions inside the membrane, hindering the ionic transport to the electrode to balance the charge changes during the redox reactions. Thus, these films behave as an inadequate electrolyte.

**Figure 10:**
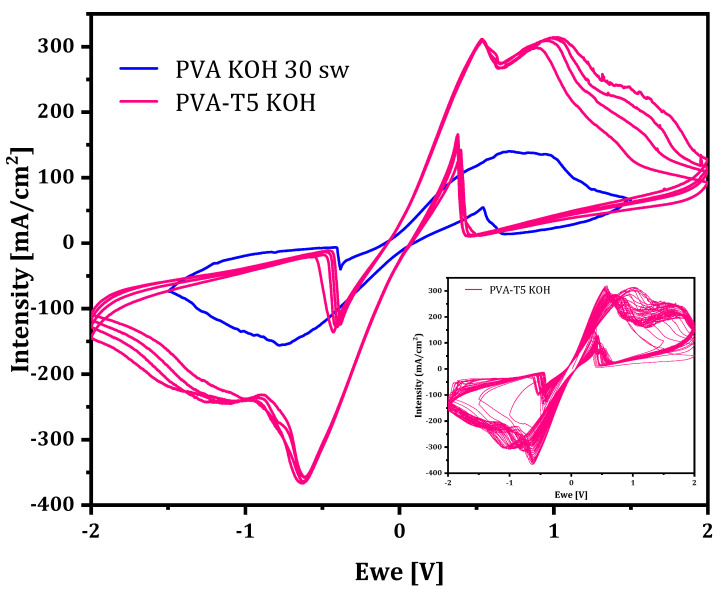
Cyclic voltammograms of the PVA-T4 KOH and PVA-KOH 30 swollen membranes [4]. Inset: 30 consecutive cycles of the PVA-T4 KOH film.

Contrarily, when PVA-VAVTD KOH GPEs (Figure 9) were used in the cell, a quasi-reversible behavior of the Zn^2+^/Zn oxidation/reduction processes was obtained. As can be seen in Figure 9 and Figure 10, two peaks (a_1_ and a_2_) are observed at the anodic branch. Similar peaks have been previously reported by Cai et al. [49] in an electrochemical study of the Zn-electrode in alkaline solution. They assigned the a_1_ peak to the oxidation of Zn to Zn(OH)_4_^2−^, whereas the peak a_2_ was associated with the oxidation of Zn to Zn(OH)_3_^–^, which is due to the depletion of (OH)^–^ anions in the proximity of the electrode surface, forming a prepassive layer at a more positive potential than that of peak a_1_. These authors only observed the double peak in the anodic branch because they used a usual three-electrode cell. However, we found the peak split in both anodic (a_1_ and a_2_) and cathodic (c_1_ and c_2_) branches because we used an Zn/PVA-VAVTD KOH/Zn cell.

Moreover, Figure 9 shows the voltammograms obtained using PVA-VAVTD KOH GPEs synthesized with different amounts of VAVTD, where the peak charges and intensities increase with the concentration of VAVTD inside the gel. This behavior agrees with increase in the swelling of the KOH solution with the amount of VAVTD, which reduces the crystallinity of the GPEs and facilitates the ion transport, as was mentioned previously. In addition, the inset in Figure 10 presents 30 consecutive cycles using a PVA-T4 KOH film, which demonstrates the GPE stability.

On the other hand, the inverse peak b’ appearing in the cathodic branch is due to the oxidation of Zn after the dissolution the passive film deposited on the Zn-electrode surface, which come off during the cathodic scan. The same happens for the peak b’’ in the anodic scan [4,49].

A comparison between PVA-KOH soaked in KOH solution and PVA-VAVTD KOH GPEs is shown in Figure 10. The current intensity of the PVA-KOH system presents a maximum close to 150 mA/cm^2^ when immersed in KOH 12 M [4]. However, this value corresponds to half of that obtained when VAVTD was added, 321 mA/cm^2^. As the intensity values result depends on the number of electrons transferred between the redox species and the electrode, which depends on the ions’ movement, the current increase may be related to the improvement of the fast-ionic motion across the electrolyte matrix. Furthermore, PVA-VAVTD KOH electrolytes are presented as an alternative to be applied in energy storage devices.

### 2.3. Zn/PVA-VAVTD KOH/Air Battery

**Figure 11:**
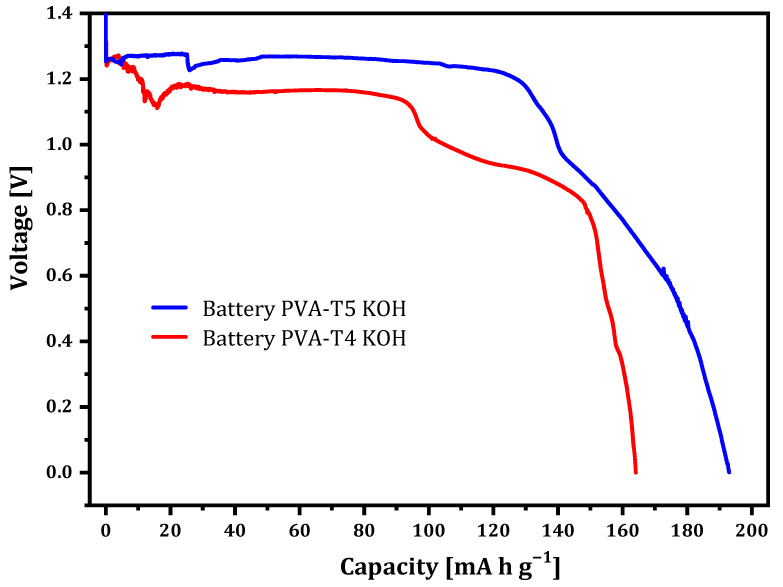
Discharge curves of Zn/PVA-T4 KOH/Air, Zn/PVA-T5 KOH/Air.

The electrochemical performances of PVA-T4 KOH and PVA-T5 KOH GPEs were examined as electrolytes in Zn–air batteries. Zn powder and a commercial Air E4B electrode were used as negative and positive electrodes. PVA-VAVTD-based membranes were used as electrolyte, placing them between Zn and air electrodes. The discharge current density was −5 mA/cm^2^ and the cut-off voltage was 0 V (Figure 11).

Zn–air batteries using PVA-VAVTD GPEs present a capacity of 135 mAh/g and 150 mAh/g for PVA-T4 KOH and PVA-T5 KOH, respectively, with a cut-off voltage of 0.9 V. However, when the discharge is carried out until a cut off of 0 V, the maximum capacity reached was 165 mAh/g and 195 mAh/g for PVA-T4 KOH and PVA-T5 KOH, respectively During the discharge process, a stable potential between 1.1 and 1.2 V until reaching 95 mAh/g was maintained when using PVA-T4 KOH GPE, whereas with PVA-T5, KOH maintains a potential of 1.2–1.3 V until reaching 150 mAh/g, confirming the positive effect of VAVTD on the discharge performance, in agreement with the cyclic voltammetry results. It is normally accepted that Zn oxidation needs enough (OH)^–^ anions to form soluble species such as Zn(OH)_4_^2–^ or Zn(OH)_3_^–^, previously to be deposited as a passive film of ZnO. Furthermore, the increase in KOH and water swelling during the immersion of the membranes in 12 M KOH solution is essential to enable the battery to function properly. Thus, these results agree with the values obtained in the CV and conductivity measurements, where high carrier charges are transported through PVA-VAVTD KOH membranes.

In addition, the best and more stable battery performance was found when the PVA-T5 KOH GPE was used. This result is in accordance with the structural characterization results, which confirmed a larger amorphous phase and higher amount of KOH solution uptake in the GPE, with the amount of VAVTD incorporated to the blend. Thus, once again, the battery results agree with the CV and conductivity measurements.

## 3. Conclusions

In this article, we proved the good electrical properties of the PVA-VAVTD blends as a consequence of the synergetic interactions of PVA and VAVTD polymers, separately improving the individual properties of each polymer. The VAVTD terpolymer has a high swelling ratio when it is soaked in 12 M KOH solution. However, their poor mechanical properties provoke its failure as a gel polymer electrolyte. This fact makes it necessary that it is blended with another polymer with better mechanical properties. For this aim, PVA was chosen due to the well-known high performance of this polymer when used as host of a GPE.

All PVA-VAVTD blends prepared present higher swelling ratios, as values of ~50–60% are obtained, than PVA-KOH polymers, which arise with maximum values of 36%, when they are soaked in KOH solution. This behavior provides a higher quantity of KOH and H_2_O molecules inside the polymeric matrix of PVA-VAVTD blends. In addition, the swelling ratio increased with the amount of VAVTD included in the membrane.

On the other hand, the structural characterization carried out by TGA, ATR-FTIR, and XRD techniques revealed the decrease in the crystallinity inside the polymer blend when VAVTD was mixed with PVA. In addition, the amorphousness of the blend raised when it was soaked in KOH solution.

Regarding the electrochemical characterization, both the conductivity and voltametric intensity values increased with the amount of VAVTD incorporated into the GPE. Ionic conductivity values demonstrate an Arrhenius behavior with the temperature and a maximum value of 0.019 S/cm at 20 °C was found, which is somewhat less than the value obtained for PVA-KOH. However, it must be noted that the Activation Energy values, ~0.077 eV, are one magnitude order lower than those measured for PVA-KOH GEPs. In addition, a quasi-reversible voltametric behavior was observed for all PVA-VAVTD blends, whose intensity peaks increase with the amount of VAVTD into the polymer and reaching intensity values approximately three times higher than those found for PVA-KOH gels, thus confirming the high ionic transfer through the gel polymer to favor the Zn/Zn^2+^ redox processes. Finally, PVA-VAVTD KOH GPEs were tested in Zn/PVA-VAVTD-KOH/air batteries, showing remarkable capacity values.

As concluding remarks, the blend of PVA and VAVTD polymers and its immersion in KOH solution provide high-performance gel polymer electrolytes, with high mechanical and electrical properties, as a consequence of the extended amorphous regions and the free volume existent inside the polymeric matrix, caused by the interaction between the VAVTD and PVA chain, as well as the high KOH and water molecules uptake. Furthermore, this GPE is a good candidate to be used in all-solid energy storage devices.

## 4. Materials and Methods

Poly (vinyl alcohol) (PVA), MOWIOL 18-88 (MW 130,000), and potassium hydroxide (KOH), Mw = 56.12 g/mol, 85% of purity were purchased from Sigma-Aldrich. Distilled water was used as a solvent in the polymers blending.

For the electrochemical characterization, zinc (Zn) powder, 98.7% purity, and Pt foils, 99.95%, were provided by Goodfellow (Hamburg, Germany), air E4B by Electric Fuel Ltd. (Beit Shemesh, Israel), and Al_2_O_3_ Polishing Suspension, (1 and 0.05) micron, Buehler (Lake Bluff, IL, USA).

### 4.1. VAVTD Synthesis

Poly (vinyl acetate)-co-poly (butyl acrylate)-co-poly (vinyl tert-decanoato) (VAVTD), of a particle size in the range of 150–300 nm [50], was synthetized with the same chemicals and procedure previously reported [29]. Vinyl acetate (Tg = 35 °C and water solubility of 2.5 g/100g at 20 °C), butyl acrylate (Tg = −53 °C and water solubility of 0.16 g/100g at 20 °C), and vinyl tert-decanoate (Tg = −3 °C and water solubility of 0.001 g/100g at 20 °C) with an initial concentration of 70/15/15 w/w, respectively, were the starting monomers of the emulsion polymerization. Ammonium persulfate and tert-butyl hydroperoxide were the radical initiators. The reaction was carried out at 353 K for 5 h.

### 4.2. Preparation of PVA-VAVTD GPEs

The polymer electrolyte was prepared by a solution casting method. One gram of PVA was dissolved in 15 mL of deionized water at 90 °C under continuous stirring for 2 h until PVA was completely dissolved. When the solution reached room temperature, different amounts of VAVTD (1,2,3,4,5) g were added under continuous stirring. Table 3 list the sample code used in the article. The mix was placed in a Petri dish and allowed to cast at ambient room temperature for 1 week. When the water was completely removed, 0.15 ± 0.05 cm-thick membranes were obtained. These films were immersed in a 12 M KOH solution for 1 day. To name the last blends, ‘KOH’ was added to the sample codes of Table 3.

### 4.3. Swelling Behavior of GPEs

For swelling ratio calculations, the samples were weighed before and after 24 h of being immersed in KOH. After that, the KOH absorbed was determined using Equation (2):

(2) $$\mathrm{SR}=\frac{W_{t}-W_{0}}{W_{0}}\;$$

where *W_t_* and *W*_0_ are the swollen gel weights and the initial sample, respectively.

### 4.4. Structural, Thermal, and Electrochemical Characterization Techniques

#### 4.4.1. ATR-FTIR and XRD Methods

Attenuated total reflectance Fourier transformed infrared spectroscopy (ATR-FTIR) measurements were obtained using a Thermo Nicolet 5700 Infrared Spectrometer (Waltham, MA, USA) in the wavenumber range of 4000–400 cm^−1^, with a resolution < 0.5 cm^−1^. X-ray diffraction patterns were collected using a Bruker D8 Advance laboratory diffractometer (Billerica, MA, USA), operated in the reflection Bragg–Brentano geometry and configured in the θ/θ mode to always maintain a horizontal sample position. The data were collected at room temperature, using Cu-Kα (λ = 1.5418 Å). The degree of crystallinity was calculated by applying Equation (3):

(3) $$X_{c}=\frac{A_{c}}{A_{T}}\times 100\%$$

where *A_c_* is the total crystallinity area and *A_T_* represents the total area of the XRD deconvoluted plot (the sum of the amorphous and crystalline area) using OriginPro software. The Gaussian function mode was employed to fit the XRD spectra.

#### 4.4.2. Thermal Analysis

Thermogravimetric analysis was performed on samples of 5–10 mg using a Mettler-Toledo TGA/DSC 1HT (Columbus, OH, USA), from room temperature up to 700 °C at a heating rate of 10 °C/min and under N_2_ atmosphere.

#### 4.4.3. Conductivity and Voltammetry Studies

**Figure 12:**
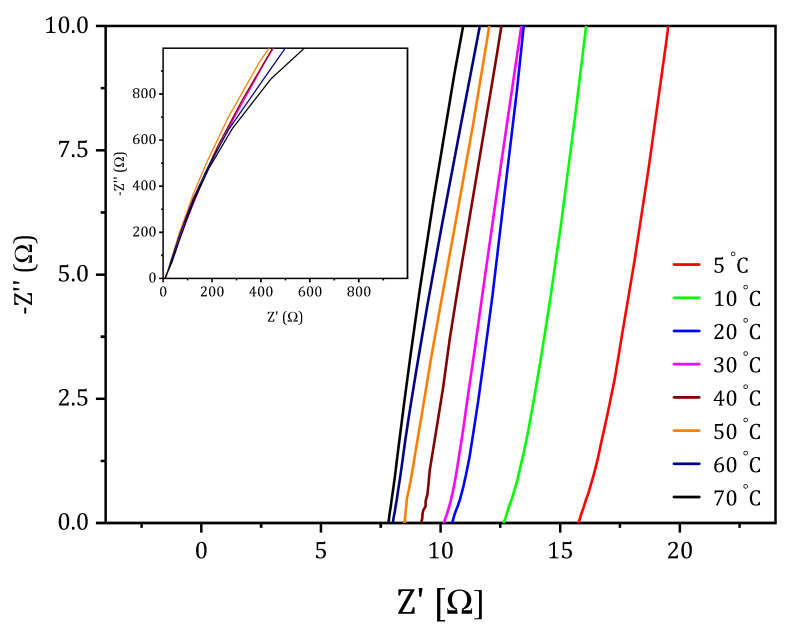
Nyquist plots obtained to calculate the bulk resistance values of the PVA-T4 KOH membrane at different temperatures.

Symmetric Zn/GPE/Zn cells were used to carried out the cyclic voltammetry measurements by a Biologic VSP Modular 5 channels potentiostat/galvanostat (Seyssinet-Pariset, France). The zinc electrodes’ area was 1 cm^2^. The scan rate was 50 mV/s, in a potential window between −2 V and +2 V. Ionic conductivity was determined by AC impedance technique using the same potentiostat/galvanostat in the frequency range from 1 kHz to 10 mHz. The sample thickness was measured with a micrometer. The temperature was set by a Julabo (Seelbach, Germany) F25-D cryothermostat in the range from 5 °C to 70 °C with a variation of ±1 °C. Two blocking platinum electrodes of 1 cm^2^ area were used in a Pt/GPE/Pt cell. Ionic conductivity, σ, was calculated using Equation (4):

(4) $$\sigma =\frac{l}{A*R_{b}}$$

where *l*, *A*, and *R_b_* represent the film thickness, the Pt electrodes area, and the bulk resistance, respectively, obtained from the intersections of impedance curves with the X axis, as shown in Figure 12. Three impedance measurements were carried out for each membrane.

To determine the activation energy (E_a_) of each electrolyte, the Arrhenius Equation (5), was used with a linear fitting by plotting a logarithmic relationship between log(σT) and 1000/T:

(5) $$\sigma =\sigma_{0}\;\exp\left(-\frac{\mathrm{Ea}}{K_{b}\left(T\right)}\right)$$

where K_b_ is the Boltzmann’s constant, T is the absolute temperature, and $\sigma_{0}$ is a pre-exponential factor [51].

### 4.5. Electrode Preparation and Battery Setup

#### 4.5.1. Zn Electrode Preparation

The electrodes were prepared with 1 g of zinc powder, which were pressed at a pressure of 10-ton cm^−2^. After that, 1 μm and 0.05 μm polishing suspensions were used to eliminate irregularities on the electrode surface. Finally, the electrodes were washed with distilled water to remove any polish trace.

#### 4.5.2. Zn/PVA-VAVTD/Air Battery Fabrication

To prepare the Zn–air batteries, the GPE was placed between 1 g of Zn powder placed in a steel capsule and the air E4B cathode. Nickel meshes were utilized as electrode collectors. Finally, the battery was placed on Teflon support to maintain constant contact during the test. Two screws located at each end of the bracket provided stability.

## Figures and Tables

**Table 1:** The *Xc* for each electrolyte calculated by the deconvoluted method.

Electrolyte	*Xc* (%)	
	Dried	Soaked in KOH
PVA	41.97	17.81
VAVTD	9.773	1.612
PVA-T1	25.48	11.90
PVA-T3	19.01	7.16
PVA-T5	15.95	5.06

**Table 2:** Swelling ratio (SR), activation energy (Ea), and the conductivity (σ) values of PVA-VAVTD KOH electrolytes. σ values were obtained at T = 20 °C.

Electrolyte	SR (%)	Ea (eV)	σ (S/cm)
VAVTD KOH	75.2	0.164	0.001
PVA-T1 KOH	52.5	0.111	0.004
PVA-T2 KOH	56.8	0.078	0.008
PVA-T3 KOH	58.6	0.077	0.011
PVA-T4 KOH	59.3	0.079	0.019
PVA-T5 KOH	62.7	-	0.019

**Table 3:** Sample code of polymer electrolytes used in this article.

Electrolyte	Sample Code
Poly (vinyl alcohol)	PVA
PVA + VAVTD (1:1)	PVA-T1
PVA + VAVTD (1:2)	PVA-T2
PVA + VAVTD (1:3)	PVA-T3
PVA + VAVTD (1:4)	PVA-T4
PVA + VAVTD (1:5)	PVA-T5

## Data Availability

No new data were created or analyzed in this study. Data sharing is not applicable to this article.
